# Supplementary material for: MC-PRPA-HLFIA Cascade Detection System for Point-of-Care Testing Pan-Drug-Resistant Genes in Urinary Tract Infection Samples
Source: Int J Mol Sci. 2023 Apr 5;24(7):6784. doi: 10.3390/ijms24076784 (PMC10095522; doi:10.3390/ijms24076784)
Supplement: Supplementary file 1 [file ijms-24-06784-s001.zip › ijms-2266433-supplementary.pdf]

Supporting Information

1. Construction of standard bacterial strains

The *E. coli* ATCC 25922 and *K. pneumonia* ATCC 13883 were received from American type culture collection. To construct the standard strains of *mcr-1*/*bla*$_{NDM}$/*tet*(X3)/*tet*(X4)/*tet*(X5)/*tet*(X6), the corresponding gene was cloned into pACYC184 and transformed into *E. coli* ATCC 25922. The *bla*$_{KPC}$ gene was also cloned into pACYC184 before being transformed into *K. pneumonia* ATCC 13883 to construct the standard strains of *bla*$_{KPC}$.

2. Enhanced RPA by PEG 200

The PEG 200 enhanced exo-RPA was administered in a 50 μL mixture containing 12.5 μL buffer A (molecular crowding reagent), 2 μL forward primer (10 μM), 2 μL reverse primer-exo (10 μM), 0.6 μL probe-exo (10 μM), 0.5 μL PEG 200, 5 μL DNA template, 24.9 μL DNase/RNase-Free water, 2.5 μL MgOAc (280 mM).

Supplementary Table

Table S1 Sequences of the primers and probes

Oligonucleotide name	Sequence (5' -3')
mcr-1 RPA F	AGTATCTTGTGGCGTGATAATAATTCGGAC
mcr-1 RPA R exo	GTCATCTAAGCCAACGAGCATACCGACATC
mcr-1 RPA R nfo	/Biotin/-GTCATCTAAGCCAACGAGCATACCGACATC
mcr-1 RPA P exo	CCGCGACCAACAACGCCATCTGCAACACCAA(FAM-dT)C(THF)T(BHQ1-dT)ATAACGAATGCCGC(C3 spacer)
mcr-1 RPA P nfo	/Digoxin/-CCGCGACCAACAACGCCATCTGCAACACCA(THF)TCCTTATAACGAATGCCGC(C3 spacer)
bla$_{NDM}$ RPA F	TCTGGCAGCACACTTCCTATCTCGACATGC
bla$_{NDM}$ RPA R exo	CCGGCAGGTTGATCTCCTGCTTGATCCAGT
bla$_{NDM}$ RPA R nfo	/Biotin/-CCGGCAGGTTGATCTCCTGCTTGATCCAGT
bla$_{NDM}$ RPA P exo	ACGGTTTGATCGTCAGGGATGGCGGCCGCG(FAM-dT)GC(THF)GT(BHQ1-dT)GGTCGATACCGCC(C3 spacer)
bla$_{NDM}$ RPA P nfo	/TAMRA/-GATGGCGGCCGCGTGCTGTTGGTCGATACC(THF)CCTGGACCGATGACCAGAC(C3 spacer)
*tet*X RPA F	CTGACTATGGCAAAATTATTACAGCAAAAC
*tet*X RPA R exo	GCAATATTTACACCCATTGGTAAGGCTAAG
*tet*X RPA R nfo	/Biotin/- GCAATATTTACACCCATTGGTAAGGCTAAG
*tet*X RPA P exo	CATAGACGTTTCAGTTTACGAAAGAGACAA [FAM-dT]G[THF]C[BHQ1-dT] GAGAGGCAAGAATTT(C3 spacer)
*tet*X RPA P nfo	/FITC/-CATAGACGTTTCAGTTTACGAAAGAGACAA(THF)GACCGAGAGGCAAGAATTT(C3 spacer)
bla$_{KPC}$ RPA F	AACCATTCGCTAAACTCGAACAGGACTTTG
bla$_{KPC}$ RPA R exo	GAAAGCCCTTGAATGAGCTGCACAGTGGGA
bla$_{KPC}$ RPA R nfo	/Biotin/-GAAAGCCCTTGAATGAGCTGCACAGTGGGA
bla$_{KPC}$ RPA P exo	ACGCGATGGATACCGGCTCAGGCGCAACTG(FAM-dT)A(THF)G(BHQ1-dT)TACCGCGCTGAGGAG(C3 spacer)
bla$_{KPC}$ RPA P nfo	/Cy3/-ACGCGATGGATACCGGCTCAGGCGCAACTG(THF)AAGTTACCGCGCTGAGGAG(C3 spacer)

Table S2 Validation of DNA extraction by modified Chelex-100 lysis method using 95 urine samples containing 10^2 CFU/mL bacteria prepared using clinically collected UTI samples

Sample ID	Double-stranded DNA (ng/μL)	OD_{260}/OD_{280}	OD_{260}/OD_{230}
1	1.78	1.17	0.32
2	1.37	1.16	0.34
3	1.43	1.17	0.32
4	1.46	1.16	0.33
5	1.21	1.16	0.32
6	1.69	1.18	0.33
7	1.55	1.17	0.32
8	1.65	1.17	0.33
9	1.55	1.18	0.50
10	1.64	1.19	0.50
11	1.39	1.15	0.42
12	1.42	1.16	0.46
13	1.67	1.17	0.49
14	1.54	1.16	0.32
15	1.72	1.18	0.31
16	1.75	1.19	0.35
17	1.62	1.17	0.42
18	1.66	1.16	0.54
19	1.52	1.17	0.41
20	1.49	1.19	0.32
21	1.57	1.19	0.35
22	1.76	1.20	0.42
23	1.62	1.15	0.33
24	1.81	1.17	0.41
25	1.52	1.15	0.44
26	1.68	1.18	0.51
27	1.06	1.21	0.33
28	1.19	1.22	0.33
29	1.06	1.21	0.33
30	1.08	1.21	0.33
31	1.02	1.25	0.33
32	1.09	1.22	0.37
33	1.07	1.23	0.36
34	1.25	1.19	0.33
35	1.28	1.25	0.33
36	1.15	1.31	0.36
37	1.19	1.24	0.33
38	1.05	1.26	0.58
39	1.14	1.24	0.30

40	1.21	1.22	0.35
41	1.09	1.20	0.32
42	1.18	1.31	0.41
43	1.13	1.22	0.46
44	1.27	1.25	0.34
45	1.16	1.30	0.35
46	1.05	1.31	0.32
47	1.44	1.21	0.33
48	1.56	1.22	0.36
49	1.55	1.21	0.33
50	1.32	1.21	0.48
51	1.22	1.23	0.32
52	1.60	1.22	0.32
53	1.58	1.22	0.33
54	1.57	1.22	0.32
55	1.58	1.23	0.36
56	1.35	1.25	0.35
57	1.56	1.27	0.37
58	1.45	1.25	0.34
59	1.36	1.23	0.30
60	1.64	1.22	0.38
61	1.72	1.24	0.34
62	1.70	1.25	0.34
63	1.42	1.30	0.32
64	1.39	1.31	0.35
65	1.46	1.20	0.31
66	1.65	1.24	0.34
67	1.53	1.25	0.36
68	1.56	1.28	0.33
69	1.60	1.25	0.39
70	1.42	1.22	0.38
71	1.59	1.31	0.32
72	1.57	1.22	0.28
73	1.46	1.22	0.32
74	1.56	1.23	0.33
75	1.64	1.22	0.33
76	1.64	1.23	0.33
77	1.60	1.26	0.32
78	1.49	1.22	0.32
79	1.46	1.25	0.33
80	1.29	1.23	0.31
81	1.58	1.25	0.34
82	1.63	1.32	0.35
83	1.59	1.23	0.40

84	1.45	1.22	0.32
85	1.48	1.30	0.31
86	1.64	1.27	0.35
87	1.61	1.29	0.38
88	1.62	1.32	0.38
89	1.54	1.27	0.34
90	1.48	1.26	0.35
91	1.47	1.23	0.38
92	1.61	1.25	0.34
93	1.68	1.29	0.35
94	1.54	1.28	0.32
95	1.49	1.21	0.33

Table S3 Detection parameters for RPA amplification and HRP-catalyzed LFIA of *mcr-1*, bla_{NDM}, bla_{KPC}, *tet*(X)

Target	RPA reaction time (min)	RPA reaction temperature (°C)	Amount of anti-biotin (HRP conjugate) (μg)	Volume of HRP-AuNPs-antibody conjugate (μL)
mcr-1	15	41	6	3
bla_{NDM}	15	43	6	5
bla_{KPC}	15	41	4	2
tet(X)	15	41	4	2

Supplementary figures

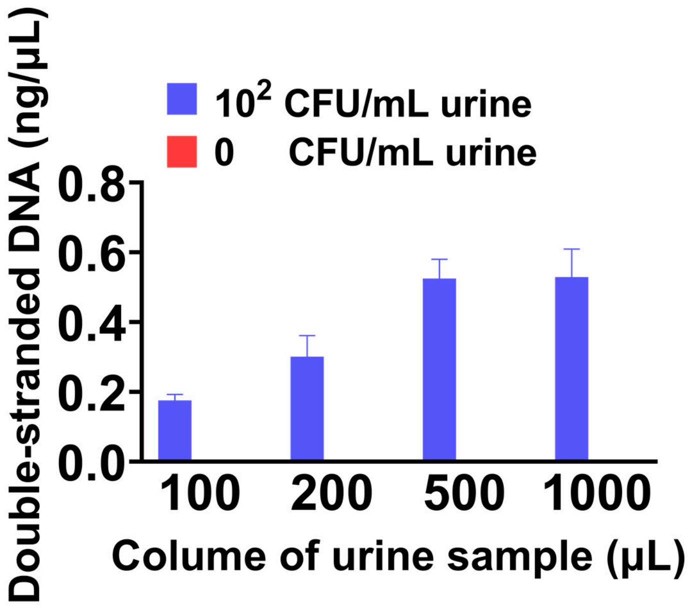

Figure S1 Determination of the volume of urine sample used for bacterial DNA extraction from bacterial-containing urine samples using the modified Chelex-100 lysis method.

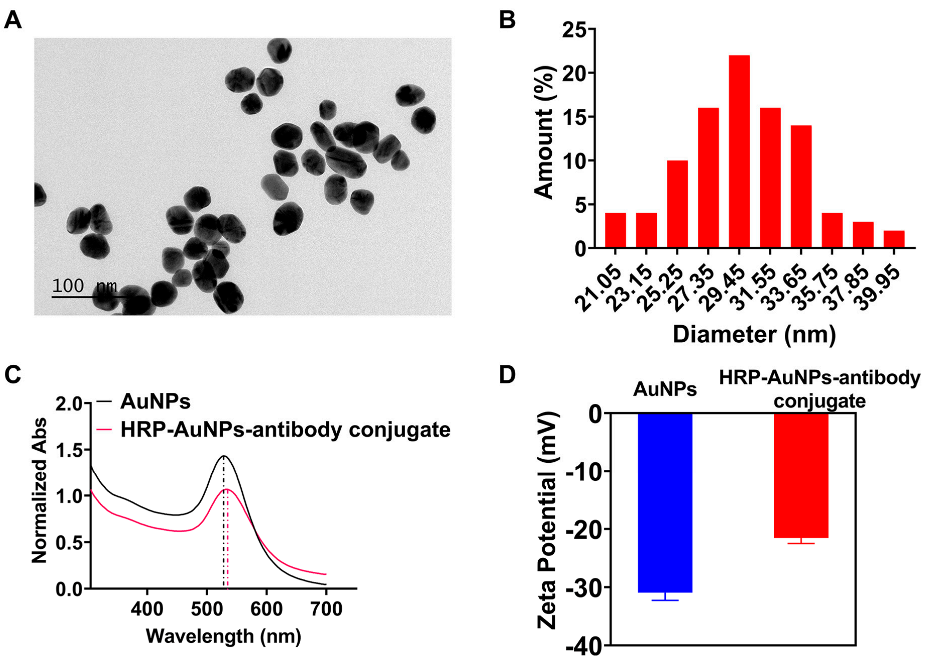

Figure S2 Characterizations of AuNPs and HRP-AuNPs-antibody conjugate. (A) TEM image of AuNPs, (B) DLS of AuNPs, (C) UV-vis spectrum of AuNPs and HRP-AuNPs-antibody conjugate, (D) Zeta potential of AuNPs and HRP-AuNPs-antibody conjugate.

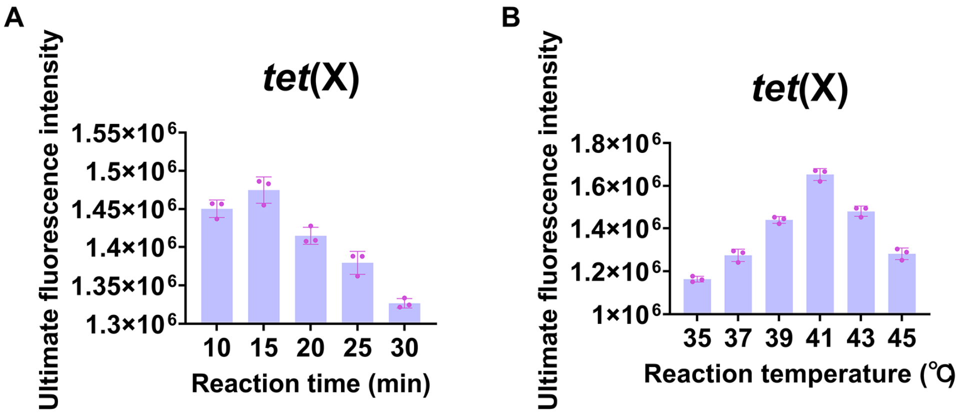

Figure S3 Optimization of PEG 200 enhanced RPA reaction conditions. (A) RPA reaction time of *tet*X, (B) RPA reaction temperature of *tet*X.

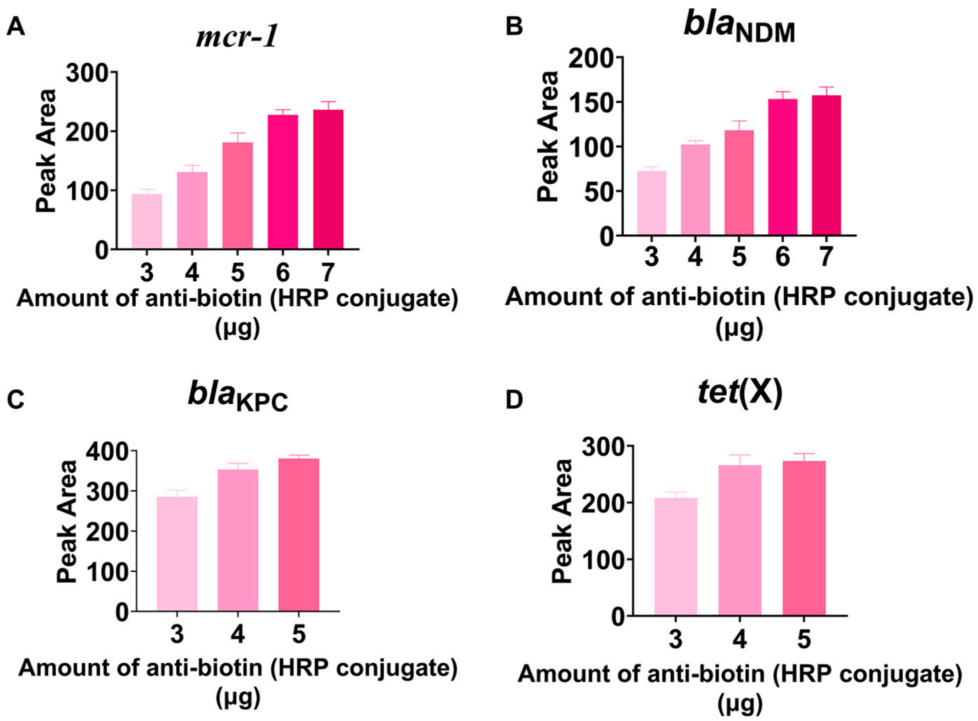

Figure S4 Optimization of the amount of anti-biotin (HRP conjugate) using in HRP-catalyzed LFIA. (A) *mcr-1*, (B) *bla*NDM, (C) *bla*KPC, (D) *tet*X.

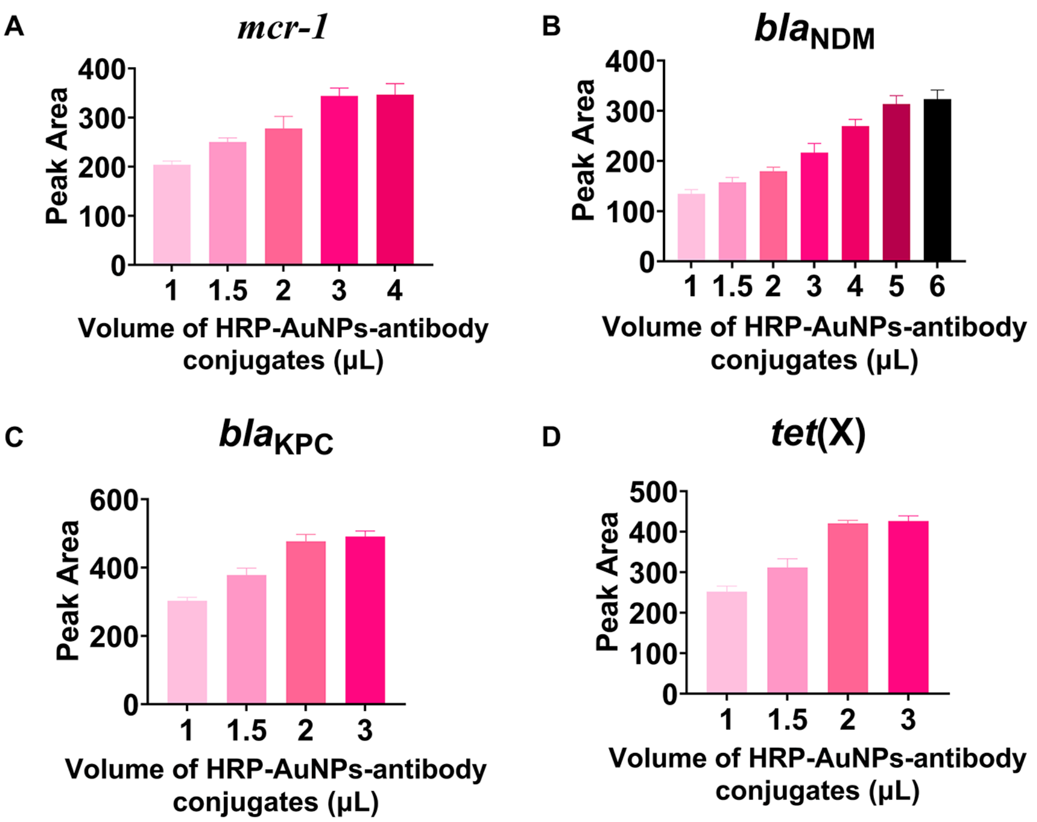

Figure S5 Optimization of the volume of HRP-AuNPs-antibody conjugate using in HRP-catalyzed LFIA. (A) *mcr-1*, (B) *bla*NDM, (C) *bla*KPC, (D) *tet*X.
